# Medical Department of the University of Buffalo

**Published:** 1854-10

**Authors:** 


### EDITORIAL DEPARTMENT.

*Medical Department of the University of Buffalo.*—The last number of the New York Journal of Medicine, has a complimentary notice of this school. A casual error in its statement of our hospital advantages, leads us to state briefly the advantages possessed by Buffalo for medical education.

The convenience of the arrangement of the college building is unsurpassed by any similar edifice in the country. The lecture rooms are large, well lighted, and furnished with a very ingenious arrangement of seats, by which each student is accommodated with an iron arm chair, the seats being ranged around the sides of the amphitheater. The museum contains a large collection of pathological and physiological specimens. In the departments of obstetrics and surgery it is particularly well furnished with material for illustration.

The Hospital of the Sisters of Charity, located immediately in the rear of the college, contains 250 beds, all of which have been occupied for some months past. The variety of disease displayed, and the facilities afforded to students for observation, are equal to those to be obtained in any school in the country. *All* the class are admitted to the clinical lectures, as the matriculation ticket covers the hospital charges.

In the department of practical anatomy no pains have been spared to furnish an abundant supply of the best material; which will be afforded at a reduction of one-half from previous prices. The dissecting rooms are large, warm, and well lighted, and afford every convenience for the pleasant prosecution of this important study.

The dissecting rooms will be opened on the 16th of October, under the charge of the Demonstrator, Dr. Boardman.

At the same time clinical lectures will be delivered at the hospital, by Profs. Hamilton and Rochester.

The regular term will commence on the first of November, and continue the usual time.

The advantages we have enumerated are not to be obtained except in large cities, and few of the eastern cities possess them in so convenient a form as they exist at Buffalo. The expenses of living in Buffalo are less than in New York or Philadelphia, and as cheap as in any considerable town. The only argument in favor of country schools is found in their cheapness; a quality, which, however desirable, is necessarily obtained at a sacrifice of clinical instruction, and of the other advantages of a city residence. The saving of twenty or fifty dollars in this manner is but a small consideration to the student who reflects that he has but two or three courses of lectures to attend to fit him for a lifetime of exertion, in which he will require all the skill which the best of advantages during student life can bestow. H.

